# Baastrup’s Disease, Interspinal Bursitis, and Dorsal Epidural Cysts: Radiologic Evaluation and Impact on Treatment Options

**DOI:** 10.7759/cureus.1449

**Published:** 2017-07-09

**Authors:** Jesse Hatgis, Michelle Granville, Robert E Jacobson

**Affiliations:** 1 Pain Management, Phoenix Neurological and Pain Institute; 2 Miami Neurosurgical Center, University of Miami Hospital

**Keywords:** spinal stenosis, lumbar epidural cysts, dorsal spinal cysts, kissing spines, radiofrequency ablation, baastrup's disease, neurogenic claudication

## Abstract

Baastrup’s disease or "kissing spines syndrome" was first described as a cause of lumbar pain before computerized tomography (CT) and magnetic resonance imaging (MRI) scanning existed. The diagnosis was based on x-ray studies, which showed that the spinous processes, especially in the lower lumbar spine, became approximated to each other and this was a generator of positional back pain. Biomechanically, the interspinous and supraspinous ligaments that are degenerated in Baastrup's disease normally contribute significantly to sagittal alignment. Ligamentous stenosis and anterolisthesis would be the expected pathology with deterioration of these ligaments and were initially described on CT and MRI in patients with symptoms similar to Baastrup's disease as isolated individual case reports. This review will highlight the relationship between the various clinical presentations, biomechanics, and overlap of Baastrup's disease with interspinous bursitis, segmental stenosis, and instability, presenting them as a disease continuum rather than as separate disease processes.

## Introduction and background

**Figure 1:**
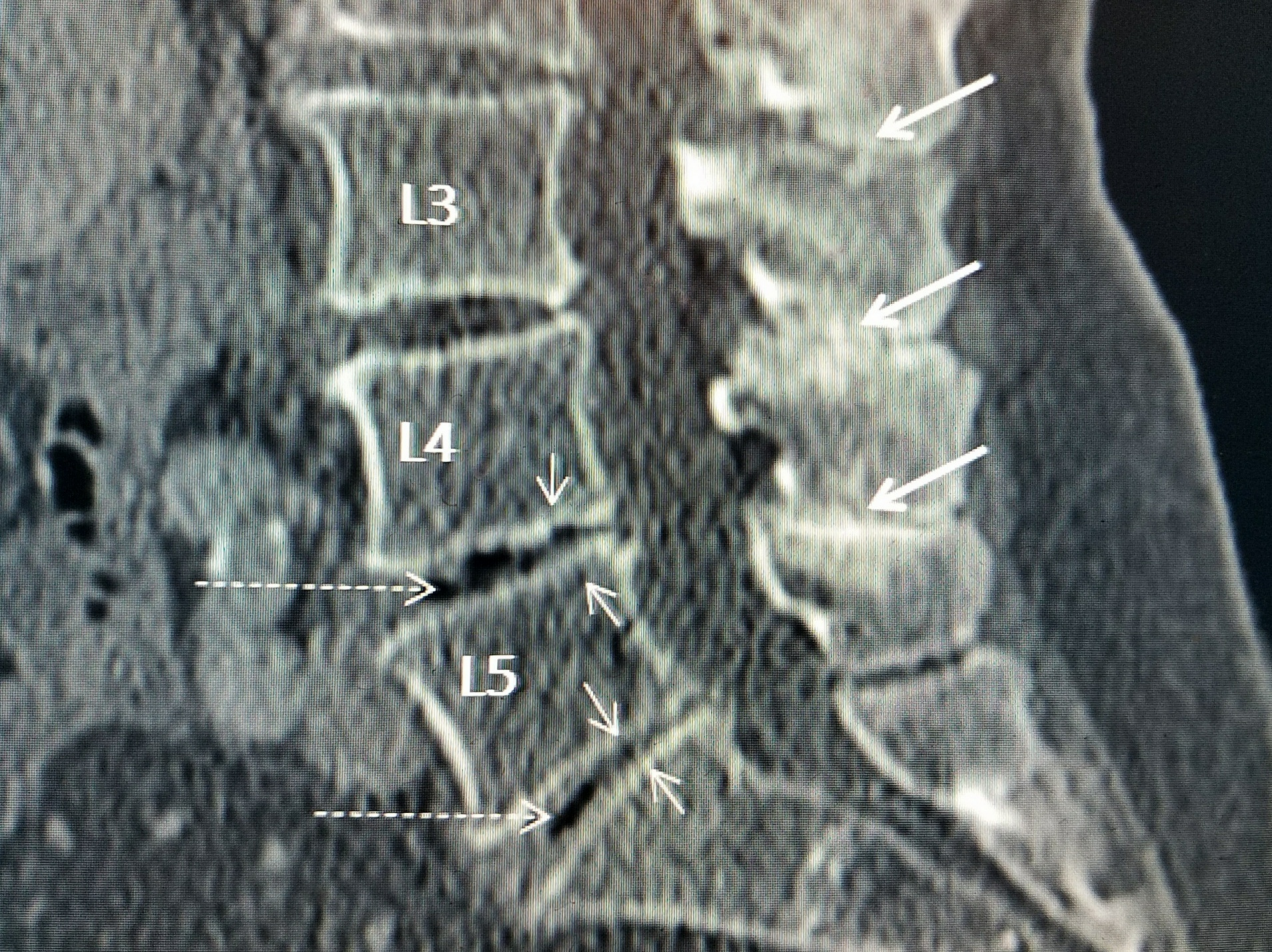
Sagittal computerized tomography (CT) scan showing 'kissing' spinous processes Sagittal lumbar CT shows the close approximation and common hyperostosis seen with the spinous processes of Baastrup's disease (thick white arrows). Also shown is associated disc space narrowing at multiple levels especially L4-5 and L5-S1 (thin white arrows) and 'vacuum' phenomenon characteristic of advanced disc degeneration (dashed white arrows) with disc necrosis and intradiscal clefts at L4-5 greater than at L5-S1.

Baastrup's disease was first described in 1933 as a cause of postural back pain, which was thought to be related to the adjacent 'kissing' of the spinous processes [1]. The diagnosis was based on 'symptoms' of positional back pain with extension, and plain radiographs showing close approximation of the spinous processes (Figure 1).

Gradually throughout the years, individual case reports in patients with clinical symptoms of Baastrup's disease were published describing ligamentous stenosis and large dorsal epidural cysts causing neurogenic claudication, as well as interspinous fluid collections believed to be related to the epidural cyst. Review of these individual case reports clearly demonstrates a spectrum of abnormalities involving weakening of the interspinous ligament that can lead to the formation of interspinous fluid clefts, facet and dorsal epidural cysts, intervertebral disc narrowing, and spinal instability with anterolisthesis and ligamentous posterior spinal stenosis [2-3]. By relating the biomechanical role of the interspinous and supraspinous ligaments and the different types of radiologic findings seen in Baastrup's disease, interspinous bursitis and segmental instability this review highlights the underlying interrelationship of what is often different stages of the same lumbar segmental degeneration.

## Review

Biomechanical studies show that the interspinous ligament works in combination with the supraspinous ligament and the ligamentum flavum to provide flexion resistance to the lumbar spine [4]. The interspinous ligament is one of the mechanisms that maintains sagittal stability of the spinal segment. Inflammatory reaction with fluid and cyst formation within the interspinous ligament can result from chronic repetitive weakening and stretching of the ligament. The ventral area near the posterior epidural space is the weaker section and where cysts develop [5]. Degeneration and weakness of this ligament leads to spinous process approximation, but more importantly a tendency to develop instability and anterolisthesis, or forward shifting of the superior vertebrae over the inferior vertebrae, at the involved segment [6]. The different pathologic changes identified on magnetic resonance imaging (MRI) scans with both Baastrup’s disease, interstitial bursitis and dorsal epidural cysts are part of the degenerative and biomechanical process that occurs after the interspinous and supraspinous ligaments deteriorate and lose tensile strength [7]. There are obviously very few pathologic studies of Baastrup's disease but bursas are commonly found in the interspinous space. In autopsies, interspinous cysts with inflammatory changes, bone erosion, and bone hypertrophy have been frequently described and are significantly more common with advanced age, being found in up to 40% of specimens [8]. 

The actual contact of the spinous processes seen on plain radiographs as the basis for diagnosing Baastrup's disease may often be a minor part of all the patient's radiologic findings and may be indicative of underlying segmental degeneration. Computerized tomography (CT) scans show bony hypertrophy of the touching spinous processes combined with reactive sclerosis as well as facet joint hypertrophy [9]. As MRI became the standard imaging technique of the spine, intraspinal cysts and other facet pathology began to be ‘associated’ with interspinous fluid bursas. There is a wide range of different bursas and cysts seen in these patients commonly identified in the facet joints. MRI scans reveal a spectrum of abnormalities in the interspinous ligament from fluid cystic change, thickening of the adjacent ligamentum flavum, and anterolisthesis of the involved vertebrae. There was often multilevel disease on MRI. Bursas and clefts were most commonly seen at L4-5, but also seen at L3-4 and L5-S1. Connections between these bursas and the epidural space have been found in 50% of the cases [9].

**Figure 2:**
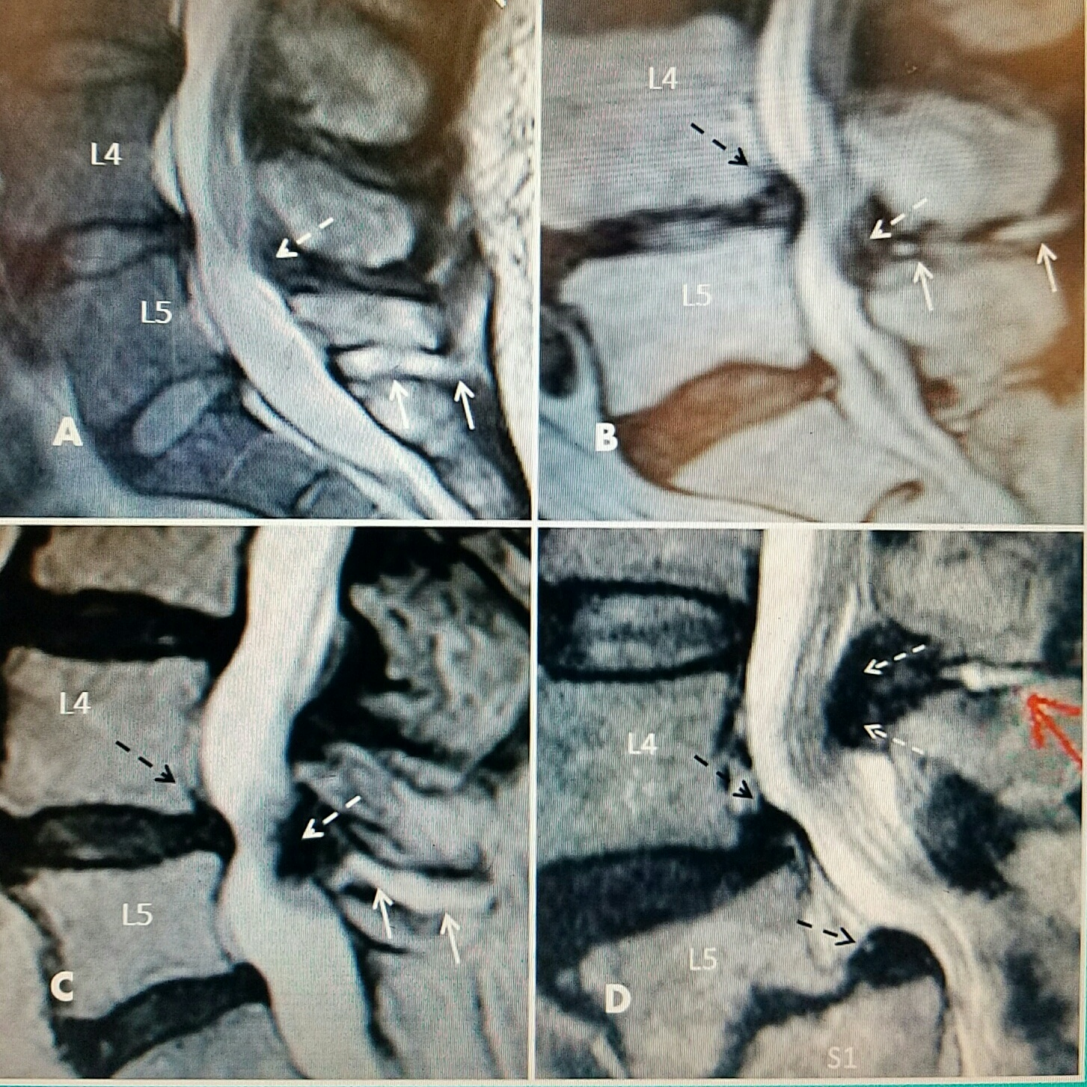
Interspinous fluid with L4-5 one level stenosis on T2 sagittal magnetic resonance imaging (MRI) studies A: L5-S1 large interspinous hyperintense fluid (two solid white arrows) with no ligamentous enlargement. L4-5 minimal ligamentous enlargement posteriorly (dashed white arrow) with early canal stenosis. L4-5 intervertebral disc has early signs of T2 signal desiccation. B: Narrowed and dessicated intervertebral disc at L4-5 with annular bulge (dashed black arrow) with minimal dorsal posterior spinal cyst (dashed white arrow) causing posterior central canal stenosis connected to interspinous fluid (solid white arrows). C: Grade 1 spondylolisthesis at L4-5 (dashed black arrow), posterior fibrous ligamentum flavum hypertrophy (dashed white arrow) and fluid in interspinous space (solid white arrows). D: Degenerated narrowed L4-5 intervertebral disc with annular bulge and grade 1 spondylolisthesis (superior dashed black arrow). Grade 2 spondylolisthesis at L5-S1 with marked endplate degeneration (inferior dashed black arrow). Posterior interspinous cyst (solid red arrow) with marked ligamentous hypertrophy and stenosis at L4-5 (two dashed white arrows). Marked edema and displacement of cauda equina roots.

Incorporating the biomechanics together with the radiologic findings, we hypothesized that repetitive shear due to weakening of the interspinous and supraspinous ligaments leads to the development of interspinal adventitious bursas and cysts, and extension of an inflammatory process within the ligamentum flavum. This progressive degenerative process contributes to the frequently observed soft tissue canal stenosis reported in many cases, although many patients present initially with localized positional lumbar pain and not neurogenic claudication (Figure 2).

Originally, due to Baastrup's description relating the problem to the spinous processes, treatment was directed to the interspinous abnormality seen on plain radiographs and originally consisted of surgery for removal of the spinous process with poor and inconsistent results. Beks reviewed 64 patients who had spinous process resection with very poor results [9]. Only 11/64 patients reported pain relief. Radiographic studies in the form of x-rays (i.e., no CT or MRI) were retrospectively reviewed and showed that all 53 of the failed surgical patients had other spinal pathology including lumbar spondylosis in 55%, disc degeneration in 23%, and spinal stenosis in 13%. He correctly attributed the unrecognized pathology to be the reason resection of the spinous process was unsuccessful. The conclusion was Baastrup's disease is not a disease entity by itself; rather it is a result of mechanical changes in the interspinous and supraspinous ligaments, degenerative lumbar discs, and facets with interspinous bursas, osteophyte formation, and spondylosis [2].

**Figure 3:**
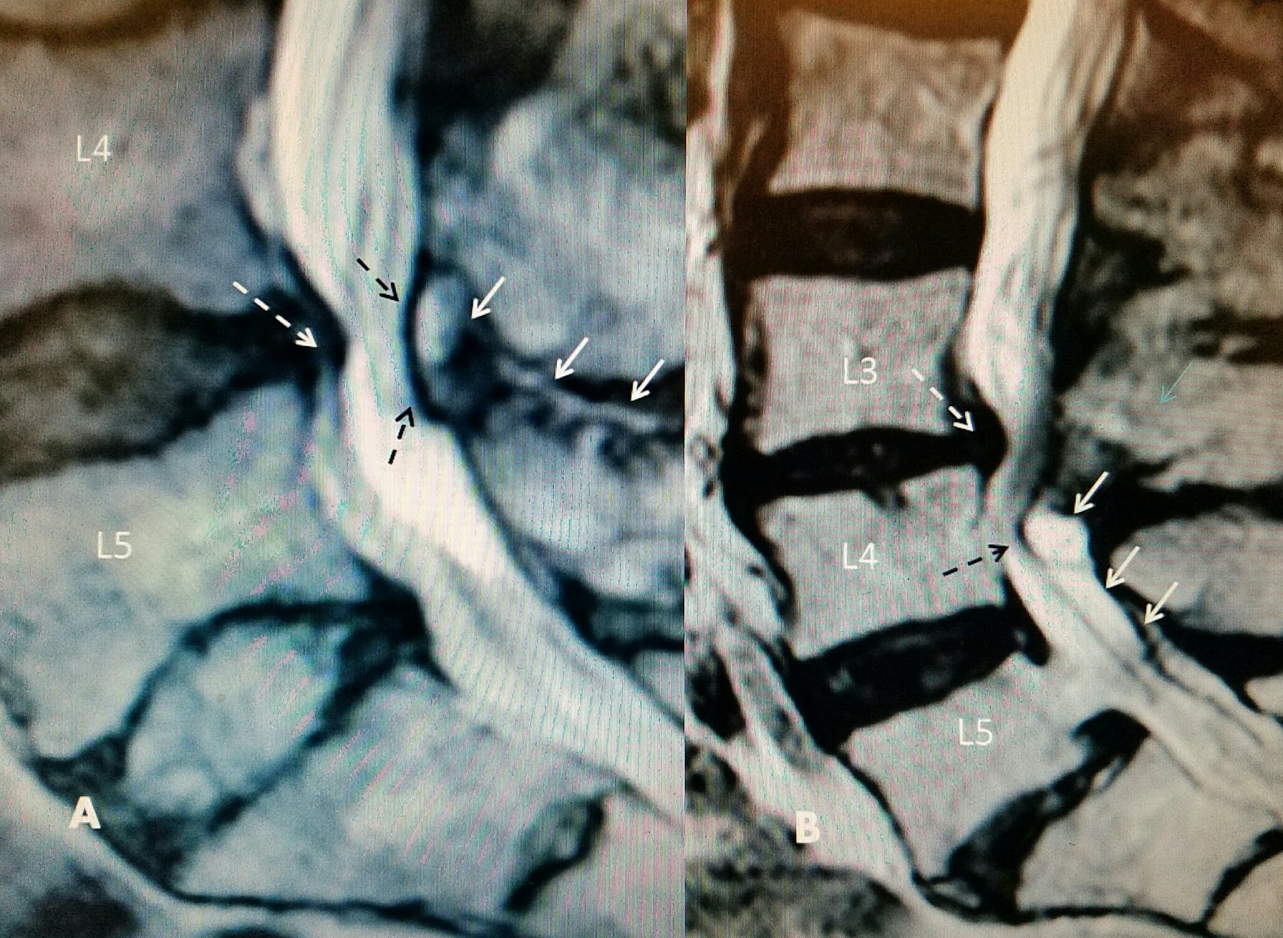
T2 sagittal magnetic resonance imaging (MRI): L4-5 disc degeneration with posterior dorsal cyst and interspinous hyperintense fluid A: Interspinous hyperintense T2 fluid signal at L4-5 (solid white arrows) leading into posterior epidural cyst causing lumbar stenosis (dashed black arrows). There is associated disc desiccation and posterior annular bulging (dashed white arrow). B: L3-4 grade 1 spondylolisthesis and annular bulge (dashed white arrow) with narrowed L3-4 disc space with large posterior cyst (uppermost solid white arrow) with T2 hyperintense signal inferior to hypertrophied ligamentum flavum (middle and lower solid white arrows). Cauda equina compression at L4 level (dashed black arrow). Separate grade 1 spondylolisthesis at L5-S1.

Initially, individual case reports were published of finding epidural cysts and masses causing stenosis associated with Baastrup’s disease, but it was later recognized that these two problems were much more commonly associated [10-11]. Chen, et al. reviewed 10 cases of posterior dorsal intraspinal cysts and noted concurrent degenerative disc disease and variable degrees of stenosis, spondylolisthesis at or below the level of the cyst with marked facet degeneration, and three patients had facet joint effusions [12]. They concluded that Baastrup's disease is associated with interspinous fluid and if the fluid bursa is large enough it could extend into the posterior epidural space causing canal stenosis. They also noted that not all cysts communicate with the facet joints or dorsal epidural space. MRI also showed severe stenosis due to a posterior non-cystic fibrous mass often with a linear fluid signal from the interspinous space. In surgery, an interspinous cleft was found and easily probed without excising the interspinous ligament. The histology reports showed the composition to be a collagen matrix mass with peripheral inflammation. They suggested that the Baastrup's cyst may change over time becoming more fibrous. Kwong, et al. performed another radiologic retrospective review of 1008 CT scans and found that 41% had evidence of Baastrup's disease, most commonly at L4-5, and the frequency of this finding increased with age and also became multilevel [13]. They concluded Baastrup's disease was part of the expected spinal degenerative changes with age and urged caution before diagnosing Baastrup's disease as the sole cause of back pain. A study with the use of interspinous distraction and stabilizing devices clearly demonstrated that stabilizing the interspinous space leads to cyst resolution and symptomatic relief, suggesting that cystic degenerative changes are a reaction to the instability resulting from deterioration and laxity of the degenerative interspinous ligament [14-15] (Figure 3).

Patients present with symptoms of either localized back pain worse with extension (i.e., typical Baastrup's disease), or more generalized back pain secondary to either facet degeneration or associated with symptoms due to spinal canal compression [3, 13, 16]. If patients are symptomatic in 'classical' Baastrup's disease they only have positional and very localized midline back pain [1-3]. It is important to determine if patients have other signs and symptoms more typical of facet degeneration, degenerative spondylolisthesis, spinal stenosis, or neurogenic claudication. If there is a failure of significant pain reduction after conservative treatments including physical therapy and anti-inflammatory medication, injections are then usually considered. Typical injections are done via fluoroscopically guided spinal needle placement between the two spinous processes and/or within the area of fluid formation seen on MRI scan with aspiration first performed (if there is cystic fluid within the interspinous space on MRI scan) and followed by a steroid injection [17-20]. If pain recurs, another option is placing a radiofrequency electrode in the same manner with thermal coagulation of multiple areas within the cyst and interspinous ligament. If there are symptoms of neurogenic claudication and MRI reveals a dorsal epidural cyst with or without ligamentous stenosis, then radiofrequency electrodes can also be used to extend the lesion into the posterior epidural space. This has been shown to decrease or ablate the posterior cyst and associated canal stenosis [21]. If the predominant symptoms are related to neurogenic claudication or radiculopathy, then surgical decompression or stabilization may be necessary [16]. In the evaluation of clinical symptoms relative to radiologic findings on CT and MRI, it is critical to not over diagnose the underlying degenerative findings [15]. If the patient has only localized postural back pain with extension without any radicular findings or claudication, then initial use of simple interspinous infiltration or radiofrequency ablation within the interspinous ligament should be the appropriate initial treatment after failing conservative measures.

## Conclusions

Degeneration within the interspinous ligament leads to loss of resistance to flexion. Better diagnostic technology demonstrates the wide range of associated cystic changes in the facet joints as well as fibrotic changes in the ligamentum flavum, which can cause symptoms of both back pain and lead to spinal stenosis with neurogenic claudication. If there is only localized back pain, treatment can be directed at the interspinous fluid or cyst with drainage and injection with corticosteroids, which is similar to treatment for lumbar facet cysts. Patients with larger cysts and especially stenosis with signs of mechanical instability may need cyst drainage or stabilization. Most importantly, the treatment ultimately needs to be focused on the underlying associated pathology of stenosis, spinal segmental instability, and/or spondylolisthesis if the patient does not benefit from simple interspinous steroid injection or there is cyst recurrence.
